# A Robust and Multi-Weighted Approach to Estimating Topographically Correlated Tropospheric Delays in Radar Interferograms

**DOI:** 10.3390/s16071078

**Published:** 2016-07-12

**Authors:** Bangyan Zhu, Jiancheng Li, Zhengwei Chu, Wei Tang, Bin Wang, Dawei Li

**Affiliations:** 1 School of Geodesy and Geomatics, Wuhan University, 129 Luoyu Road, Wuhan 430079, China; jcli@sgg.whu.edu.cn (J.L.); binwangsgg@whu.edu.cn (B.W.); dwli@sgg.whu.edu.cn (D.L.); 2 Nanjing Institute of Surveying, Mapping and Geotechnical Investigation, Nanjing 210019, China; zby7edgar@126.com; 3 State Key Laboratory of Information Engineering in Surveying, Mapping and Remote Sensing, Wuhan University, Wuhan 430079, China; weitang@whu.edu.cn

**Keywords:** InSAR, tropospheric delay, least squares, robust estimation, persistent scatterer interferometry

## Abstract

Spatial and temporal variations in the vertical stratification of the troposphere introduce significant propagation delays in interferometric synthetic aperture radar (InSAR) observations. Observations of small amplitude surface deformations and regional subsidence rates are plagued by tropospheric delays, and strongly correlated with topographic height variations. Phase-based tropospheric correction techniques assuming a linear relationship between interferometric phase and topography have been exploited and developed, with mixed success. Producing robust estimates of tropospheric phase delay however plays a critical role in increasing the accuracy of InSAR measurements. Meanwhile, few phase-based correction methods account for the spatially variable tropospheric delay over lager study regions. Here, we present a robust and multi-weighted approach to estimate the correlation between phase and topography that is relatively insensitive to confounding processes such as regional subsidence over larger regions as well as under varying tropospheric conditions. An expanded form of robust least squares is introduced to estimate the spatially variable correlation between phase and topography by splitting the interferograms into multiple blocks. Within each block, correlation is robustly estimated from the band-filtered phase and topography. Phase-elevation ratios are multiply- weighted and extrapolated to each persistent scatter (PS) pixel. We applied the proposed method to Envisat ASAR images over the Southern California area, USA, and found that our method mitigated the atmospheric noise better than the conventional phase-based method. The corrected ground surface deformation agreed better with those measured from GPS.

## 1. Introduction

When microwave signals such as Interferometric Synthetic Aperture Radar (InSAR) travel through the atmosphere, they are affected by spatio-temporal variations in the atmospheric refraction [1,2], which can result in a phase advance or delay. These atmospheric noises typically come from two major sources: ionospheric and tropospheric effects. Spatio-temporal variations in density of the free electrons found in the ionosphere (≥60 km) cause phase advances that are more significant at higher latitudes and for larger wavelength radar signals such as the L-band SAR sensor onboard the ALOS satellite [3]. Tropospheric effects are caused by spatio-temporal variations in pressure, temperature, and water vapor content in the lower part of the troposphere (≤5 km), which causes phase delays that can be up to a few decimeters in magnitude and often overwhelm the deformation signal of interest [4]. Our study only focuses on the tropospheric delay as the ionospheric effects can be neglected in the C-band SAR images that we used in our study.

A number of methods have been developed to correct the tropospheric delay in InSAR data [5,6,7]. These correction techniques can be classified into two groups: methods based on auxiliary data and methods based on the interferometric phase itself. The methods producing synthetic delay maps by using auxiliary data sets directly to correct the tropospheric delay in interferograms. This includes the use of GPS wet delay measurements [8,9,10], local meteorological data [11], multispectral remote sensing imagery [12,13], and local or global meteorological reanalysis products [14,15]. Ground-based GPS measurements are typically sparse, while multispectral remote sensing imagery such as MODIS and MERIS can only provide measurements during daytime and has a low accuracy in the presence of clouds. The local or global meteorological models have large grid spacing; and are not sufficient for simulating smaller scale tropospheric effects such as those associated with turbulent mixing [15]. In addition, the atmospheric models are not always available at the acquisition time for each SAR image.

The phase-based methods are independent of external data sets that can be used to calculate the tropospheric phase delay from the interferogram itself. Tropospheric phase delay Δ*φ_trop_* for an individual interferogram can be computed by assuming a linear model (Δ*φ_trop_* = *Kh* + *φ_c_*)), between the elevation h and interferometric phase Δϕ. Where the elevation h can be derived from the SRTM DEM, *K* is the phase-elevation ratio to be estimated, and $\phi_{c}$ represents a shift applied to the whole interferogram. The linear model has been used to correct interferograms with success in small and non-deforming areas, but it is limited, as it assumes no spatial variability in the tropospheric properties of the interferogram [16,17,18]. The power-law model ($\Delta \phi_{trop}=K^{\prime }{\left(h_{0}-h\right)}^{\alpha }$) developed by Bekaert et al. accounts for the spatially variable signal of the tropospheric phase delay [19]. The two input parameters $h_{0}$ and α are estimated from balloon sounding data or weather model data, which are not always available for a study area. Another way to mitigate the tropospheric delay is to apply spatio-temporal filtering in time series analysis based on the assumption that the delay is random in time. Due to seasonal changes in the atmospheric conditions, delay sometimes is significantly correlated with time; thus spatio-temporal filtering usually used for extracting and removing tropospheric delay will not work properly. In our study, we propose a method to estimate the phase-elevation ratio *K* in the linear model by using a robust and multi-weighted approach. Our method accounts for the spatial variability of the tropospheric phase delay by decomposing the interferogram into blocks and robustly estimating the *K* value for each block. Compared to existing methods, our approach is relatively simple and is not dependent on the availability of external data sets, and corrects the interferograms on a scene-by-scene basis.

Robust estimation has been widely used for gross error processing in least square adjustment (LS) [20,21]. This method is generally easier to perform and more efficient than data snooping, especially when there are a large number of observations. Thus, it is applicable to the estimation of the ratio *K* in the phase-elevation linear model for tropospheric correction in radar interferograms. In this paper, we introduce a robust estimation algorithm to derive topographically correlated tropospheric delays and propose a new expanded form to solve the problems caused by a massive number of InSAR pixel observations (interferometric unwrapped phases). We propose a complete solution to estimate tropospheric delays that can be applied locally to account for the spatial variability found in the troposphere. We apply our Robust and Multi-Weighted (RMW) approach to correct the interferograms over Southern California for a small area containing the Pomona basin area that has suffered from rapid subsidence due to water withdrawal.

## 2. InSAR Data Set and Interferometric Processing

**Figure 1:**
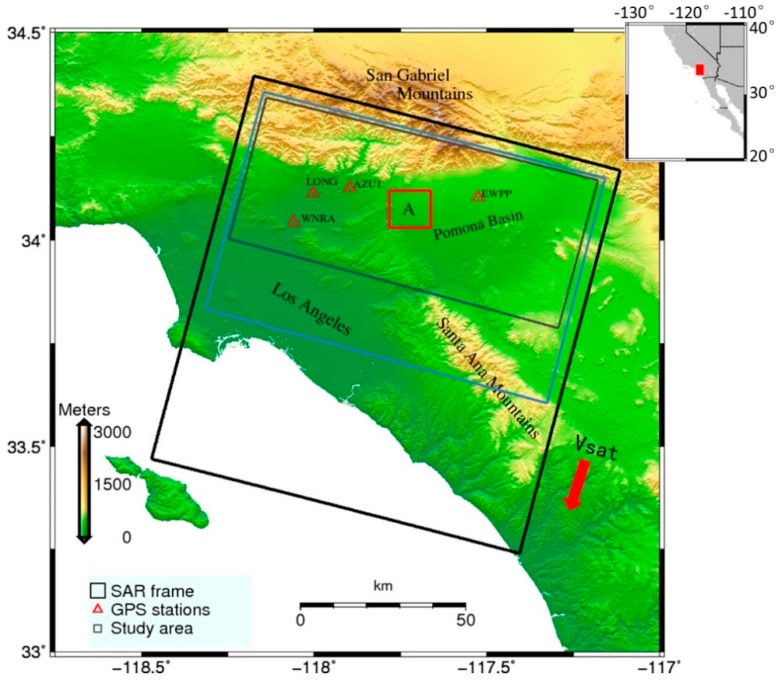
Data coverage for the study region. The area in black rectangle is the footprint of the whole Envisat image. The gray rectangle represents our study area and an experimental region A is denoted by red rectangle. The wider study area used to test turbulent effects is denoted by the blue rectangle. The red triangles represent the locations of GPS sites. The background is the SRTM DEM.

To investigate the correction capability of the RMW method, we generated a single master network of 19 descending Envisat ASAR images over Southern California, USA (track 170, see Figure 1), spanning the period from 23 February 2008 until 25 September 2010. The image acquired on 18 April 2009 was selected as the common master image. From the common master image and remaining images, 18 differential interferograms were generated. The spatio-temporal baseline parameters of these interferograms are given in Table 1.

DORIS software was used for interferometric processing and StaMPS (Stanford Method for Persistent Scatterers) software was applied to select persistent scatterer (PS) and estimate DEM errors [22,23,24]. The parameters used in the StaMPS processing are listed in Table 2. We used the precise ODR orbits from Delft Institute for Earth-Oriented Space Research to minimize the orbital errors, while the Shuttle Radar Topography Mission (SRTM) digital elevation model with 90 m resolution was used for removing topographic phase contributions [25].

## 3. The RMW Tropospheric Correction Method

### 3.1. Modeling

Tropospheric delay is related to the spatial and temporal variations in air temperature, pressure, and relative humidity in the troposphere, which can be split into hydrostatic, wet and liquid components. The wet component is the major limiting factor due to the highly unstable water vapor content in both in space and time [26,27]. Unlike the wet component, temperature and pressure are smoothly distributed in space, leading to better-resolved longer-scale wavelength hydrostatic components, which are quite stable and create about 90 percent of the total tropospheric delay. The liquid component will only become significant when the atmosphere is saturated and is expected to be quite small. This component is responsible for less than 0.1–0.4 mm/km tropospheric delay under usual weather conditions for C-band sensors (wavelength of approximately 6 cm) [28]. We focus only on combination of the hydrostatic and wet delay, neglecting the impact of the liquid water content and ionospheric electronic content (typically very small for C-band SAR data) [3]. The tropospheric phase delay Δ*φ_trop_* for an individual interferogram can be estimated from the relationship between the interferometric phase and topography:

(1) $$\Delta \phi_{trop}=Kh+\phi_{c}$$

where *K* is a constant in the phase-elevation ratio relating the interferometric tropospheric phase delay Δ*φ_trop_* and the elevation h. The value *K* is used to calculate the tropospheric signal throughout the full interferogram, and $\phi_{c}$ represents a shift applied to the whole interferogram.

However, there are two limitations in this conventional linear model (Equation (1)). First, a single relationship between phase and topography for the whole interferogram cannot account for the spatially variable tropospheric delay and second, a non-deforming region is required. In order to account for the lateral variability of the tropospheric delay, the interferograms were split into multiple blocks and the phase-elevation ratios were estimated locally for each block. The original interferometric phases are comprised of multiple signals, such as tropospheric delay, deformation, and residual orbit errors. The wavelengths of these components contain a multi-scale dependent spectrum and have different sensitivities to each other. For example, tropospheric signals contain short wavelength scale components, introduced by turbulence component in the lower part of the troposphere (≤5 km). The long wavelength scale components introduced by hydrostatic and wet delays are partly correlated to topography [28]. In addition, the longer wavelength scale components introduced by ground deformation such as tidal loading [29], tectonic slow slip [30], or hydrological loading [31] may be more sensitive to other different sources of confounding noises. Therefore, the selection of the band filter is not always trivial, as it requires empirical information about multi-scale dependent spectrums. However, we can take advantage of the fact that the tropospheric signal is present at all wavelength scales so we can estimate the phase-elevation ratio from a spatial frequency band relatively insensitive to the other signals [18]. We take consideration of the different spatial bands performance of final tropospheric results overall in our study. Based on this analysis, a 2–16 km spatial band was chosen over our test-region, the details are discussed in Section 4. To do this, we did not need a non-deforming region to estimate the phase-elevation ratio.

As mentioned above, we can rewrite the Equation (1):

(2) $$\Delta \phi_{trop,filt}=K^{\prime }h_{filt}+\phi_{c}$$

(3) $$\Delta \phi_{trop}=K^{\prime }h+\phi_{c}^{\prime }$$

where $K^{\prime }$ is a phase-elevation ratio, relating the estimated tropospheric delay based on the band-filtered phase Δ*φ_trop,filt_* and the elevation *h_filt_*. The value $\phi_{c}$ represents a constant bias term, and $\phi_{c}^{\prime }$ represents a shift applied to the whole interferogram, estimated over multiple blocks.

To estimate the phase-elevation ratio $K^{\prime }$, we estimate this value for each block locally and then these values are multiply-weighted and extrapolated to each PS point. The local robust estimation of the phase-elevation ratio plays a critical role in increasing the accuracy of tropospheric delays. We applied an expanded form of the robust estimation method; the details of the derivational processes and our corresponding strategies for data processing are discussed in the following subsections.

### 3.2. Basic Principles of the Least Squares and Robust Estimation Method

The error equation of a linear observation model is presented as:

(4) $$V=A\hat{X}-L$$

where ***L*** denotes the *m* × 1 observation vector, ***A*** represents the *m* × *n* coefficient matrix, and Rank(A)=n<m. $\hat{X}$ denotes the parameter vector to be estimated and ***V*** is the residual vector of ***L***.

The adjustment principle of least squares is as below:

(5) $$V^{T}PV=\min$$

where ***P*** denotes the weight matrix of ***L***. By solving the extreme value problem, the parameter estimator is obtained as follows:

(6) $$\hat{X}={\left(A^{T}PA\right)}^{-1}A^{T}PL=N^{-1}C$$

where ***N*** = ***A***^T^***PA***, ***C*** = ***A***^T^***PL***.

When the observation vector ***L*** contains gross errors, it is difficult to derive a reliable parameter solution using least squares method. Therefore, a robust estimation method based on the equivalent weight principle is introduced to solve this problem; the estimation criterion is presented as:

(7) $$V^{T}\bar{P}V=\min$$

where $\bar{P}$ is the equivalent weight matrix of the observation vector ***L***. The parameter estimator can be easily derived as follows:

(8) $$\hat{X}={\left(A^{T}\bar{P}A\right)}^{-1}A^{T}\bar{P}L$$

We assume that observations are independent from each other, thus the *i*th diagonal element of $\bar{P}$ is obtained as:

(9) $${\bar{p}}_{i}=p_{i}w_{ii}$$

where $w_{ii}$ represents the corresponding weight factor. We use the weight factor function of IGGIII [32,33] to calculate $w_{ii}$:

(10) $$w_{ii}=\{\begin{array}{cc} 1.0 & |{\tilde{v}}_{i}|\;\leq \;k_{0}\\ \frac{k_{0}}{\left|{\tilde{v}}_{i}\right|}{\left(\frac{k_{1}-|{\tilde{v}}_{i}|}{k_{1}-k_{0}}\right)}^{2} & k_{0}<\;|{\tilde{v}}_{i}|\;\leq k_{1}\\ 0 & k_{1}<\;|{\tilde{v}}_{i}| \end{array}$$

where ${\tilde{v}}_{i}$ represents the *i*th standardized residual, *k*_0_ and *k*_1_ are constants and can be set as *k*_0_ = 2.0–3.0 and *k*_1_ = 4.0–8.0, these values are set according to the specific application backgrounds. The value for ${\tilde{v}}_{i}$ is calculated as:

(11) $${\tilde{v}}_{i}=\frac{v_{i}}{\sigma_{0}\sqrt{qv_{i}}}$$

where *v_i_* denotes the *i*th element in the residual vector ***V***, $\sigma_{0}$ is the square root of the variance component. *qv_i_* indicates the *i*th diagonal element of ***QV***, ***QV*** is the cofactor matrix of LS residuals, and it can be derived via cofactor propagation as below:

(12) $$QV=P^{-1}-A{\left(A^{T}PA\right)}^{-1}A^{T}=P^{-1}-AN^{-1}A^{T}$$

The estimator of $\sigma_{0}$ obtained from the traditional formula is easily affected by gross errors; thus, the median method [34] is applied to obtain a robust estimator of $\sigma_{0}$. The formula is presented as follows:

(13) $${\hat{\sigma }}_{0}=\overset{m}{\underset{i\;=\;1}{med}}\left(\left|v_{i}/\sqrt{qv_{i}}\right|\right)\cdot 1.4826$$

The iterative procedure for robust estimation is described as below:

1. *Step 1*: Set the initial value of the equivalent weight matrix: ${\bar{P}}^{\left(0\right)}=P$.
2. *Step 2*: Calculate the parameter and residual vectors as follows:
  (14) $${\hat{X}}^{\left(k\right)}={\left(A^{T}{\bar{P}}^{\left(k\right)}A\right)}^{-1}A^{T}{\bar{P}}^{\left(k\right)}L$$
  (15) $$V^{\left(k\right)}=A{\hat{X}}^{\left(k\right)}-L$$
3. *Step 3*: Compute standardized residuals as below:
  (16) $$\sigma_{0}^{\left(k\right)}=1.4826*\overset{m}{\underset{i\;=\;1}{med}}\left(\left|v_{i}^{\left(k\right)}/\sqrt{qv_{i}}\right|\right)$$
  (17) $${\tilde{v}}_{i}^{\left(k\right)}=v_{i}^{\left(k\right)}/\left(\sigma_{0}^{\left(k\right)}\sqrt{qv_{i}}\right)$$
4. *Step 4*: Calculate weight factors by substituting Equations (17) into (10) and then update the equivalent weight: ${\bar{p}}_{i}^{\left(k\;+\;1\right)}=p_{i}w_{ii}$.
5. *Step 5*: Increase *k* = *k* + 1, repeat Step 2 to 4 until $\left\|{\hat{X}}^{\left(k+1\right)}-{\hat{X}}^{\left(k\right)}\right\|<\varepsilon_{0}$.

After the parameters are estimated, we can assess the precision requirements of the solution for robust estimation and the corresponding variance factor of the unit weight is estimated as:

(18) $${\hat{\sigma }}_{0}^{2}=\frac{V^{T}\bar{P}V}{m-n-n0}$$

where *n*0 represents the number of observations that contain gross errors where the weight factors are equal to 0.

Thus, the covariance matrix of the estimates can be obtained via variance propagation to Equation (8) as:

(19) $$D_{\hat{X}}={\hat{\sigma }}_{0}^{2}{\left(A^{T}\bar{P}A\right)}^{-1}$$

### 3.3. Expanded Forms of the Involved Matrices and a Test Case

A large number of observations consume a lot of memory when constructing the matrices P and $\bar{P}$. As a result, the computational burden is too large; sometimes calculation failure may occur.

In order to solve this problem, we make full use of matrix calculation principles. Since we assume that P and $\bar{P}$ are diagonal matrices, Equation (12) can be written as:

(20) $$\begin{array}{cc} QV & =\left[\begin{array}{cccc} 1/p_{1} & & & \\ & 1/p_{2} & & \\ & & \ddots & \\ & & & 1/p_{m} \end{array}\right]-\left[\begin{array}{c} A_{1}\\ A_{2}\\ \vdots \\ A_{m} \end{array}\right]\cdot N\cdot \left[\begin{array}{cccc} A_{1}^{T} & A_{2}^{T} & \cdots & A_{m}^{T} \end{array}\right]\\ & =\left[\begin{array}{cccc} 1/p_{1} & & \\ & 1/p_{2} & & \\ & & \ddots & \\ & & & 1/p_{m} \end{array}\right]-\left[\begin{array}{cccc} A_{1}NA_{1}^{T} & A_{1}NA_{2}^{T} & \cdots & A_{1}NA_{m}^{T}\\ A_{2}NA_{1}^{T} & A_{2}NA_{2}^{T} & \cdots & A_{2}NA_{m}^{T}\\ \vdots & \vdots & \ddots & \vdots \\ A_{m}NA_{1}^{T} & A_{m}NA_{2}^{T} & \cdots & A_{m}NA_{m}^{T} \end{array}\right] \end{array}$$

where ***A****_i_* represents the *i*th row of matrix ***A***. The *i*th diagonal element of ***Qv*** (*qv_i_*) therefore can be expressed as the following:

(21) $$qv_{i}=1/p_{i}-A_{i}N^{-1}A_{i}^{T}$$

The matrix ***N*** in Equations (6), (12) and (21) can be expanded as below:

(22) $$N=A^{T}PA=\left[\begin{array}{cccc} \sum_{k\;=\;1}^{m}A_{k1}p_{k}A_{k1} & \sum_{k\;=\;1}^{m}A_{k1}p_{k}A_{k2} & \cdots & \sum_{k\;=\;1}^{m}A_{k1}p_{k}A_{kn}\\ \sum_{k\;=\;1}^{m}A_{k2}p_{k}A_{k1} & \sum_{k\;=\;1}^{m}A_{k2}p_{k}A_{k2} & \cdots & \sum_{k\;=\;1}^{m}A_{k2}p_{k}A_{kn}\\ \vdots & \vdots & \ddots & \vdots \\ \sum_{k\;=\;1}^{m}A_{kn}p_{k}A_{k1} & \sum_{k\;=\;1}^{m}A_{kn}p_{k}A_{k2} & \cdots & \sum_{k\;=\;1}^{m}A_{kn}p_{k}A_{kn} \end{array}\right]$$

In addition, the matrix ***C*** in Equation (6) can be expanded as:

(23) $$C=A^{T}PL=\left[\begin{array}{c} \sum_{k\;=\;1}^{m}A_{k1}p_{k}L_{k}\\ \sum_{k\;=\;1}^{m}A_{k2}p_{k}L_{k}\\ \vdots \\ \sum_{k\;=\;1}^{m}A_{kn}p_{k}L_{k} \end{array}\right]$$

Thus, the any one element of ***N*** is summarized as:

(24) $$N_{ij}=\sum_{k\;=\;1}^{m}A_{ki}p_{k}A_{kj},\;(i=1,\cdots n,\;j=1,\cdots n)$$

The *i*th element of ***C*** is written as:

(25) $$C_{i}=\sum_{k\;=\;1}^{m}A_{ki}p_{k}L_{k},\;(i=1,\cdots n)$$

The matrices $A^{T}\bar{P}A$ and $A^{T}\bar{P}L$ have similar expanded forms with ***N*** and ***C***, respectively. We can present them as follows:

(26) $${\left(A^{T}\bar{P}A\right)}_{ij}=\sum_{k\;=\;1}^{m}A_{ki}{\bar{p}}_{k}A_{kj},\;(i=1,\cdots n,\;j=1,\cdots n)$$

(27) $${\left(A^{T}\bar{P}L\right)}_{i}=\sum_{k\;=\;1}^{m}A_{ki}{\bar{p}}_{k}L_{k},\;(i=1,\cdots n)$$

The quadratic sum $V^{T}\bar{P}V$ in Equation (18) can also be written as:

(28) $$V^{T}\bar{P}V=\sum_{i\;=\;1}^{m}{\bar{P}}_{i}v_{i}^{2}$$

Based on these expanded formulas, there is no need to program the matrices ***P*** and $\bar{P}$ and unnecessary calculations are avoided. The robust estimation method can easily be applied in cases with a large number of observations. Especially in instances when interferograms are at high resolution and with good coherence, the high density of PS pixels results in higher computational efficiency. Therefore, we can estimate the phase-elevation ratio $K^{\prime }$ locally over each block by applying the expanded forms of the robust estimation method. According to the rewritten linear model (Equation (2)), the corresponding matrices can be constructed as the following:

(29) $$\Delta \phi_{trop,filt,i}+v_{i}=K^{\prime }h_{filt,i}+\phi_{c}$$

where $\Delta \phi_{trop,filt,i}$, $v_{i}$ and $\phi_{c}$ indicate the band filtered phase, the random error, and the bias term for *i*th PS point on a local block, respectively. In the application of our robust estimation method to solve this linear model, matrices in Equation (23) are constructed as:

(30) $$A=\left[\begin{array}{cc} h_{filt,1} & 1\\ h_{filt,2} & 1\\ \vdots & \vdots \\ h_{filt,N} & 1 \end{array}\right]\;L=\left[\begin{array}{c} \Delta \phi_{\mathrm{trop},\mathrm{filt},1}\\ \Delta \phi_{\mathrm{trop},\mathrm{filt},2}\\ \vdots \\ \Delta \phi_{\mathrm{trop},\mathrm{filt},N} \end{array}\right]\;\hat{X}=\left[\begin{array}{c} K^{\prime }\\ \phi_{c} \end{array}\right]\;P=I_{N}$$

where $I_{N}$ represents the N×N unit matrix and the N indicates the number of PS on local block.

In order to verify the feasibility and advantages of our robust estimation method, we tested the approach on InSAR data from an experimental region about 100 km^2^ in size, the dataset is shown in Table 1. In the experimental region A (red rectangle in Figure 1), a small-scale deformation area located at Pomona appears to reflect water withdrawal. We estimated the local phase-elevation ratio assuming the linear model (Equation (1)) with conventional least squares method and the robust estimation method, respectively. Table 3 shows the improvement in the standard deviation values after applying our robust estimation method. These results suggest that this robust estimation method yields the phase-elevation ratio more robustly and reliably.

### 3.4. The Multi-Weighted Phase-Elevation Ratio for PS

Our Robust Multi-Weighted (RMW) method splits the study region into multiple blocks (40 in the study), which are increasing bottom up and starting in the lower left corner. To ensure adjacent consistence, these blocks within a tropospheric region constrain the phase-elevation ratio estimation with a 50 percent overlap. Then, all local ratios are multiply weighted and interpolated to all PS points for a full interferogram. The weight function is constructed using the block uncertainty of the estimate and the distance from the block centers to each PS point. Equations (31)–(33) show the form of the weight combination and final derived phase-elevation ratios for all PS points.

(31) $$S={\left[\begin{array}{cccc} s_{1}^{-1}/\sum_{i\;=\;1}^{n}s_{i}^{-1} & s_{2}^{-1}/\sum_{i\;=\;1}^{n}s_{i}^{-1} & \cdots & s_{n}^{-1}/\sum_{i\;=\;1}^{n}s_{i}^{-1}\\ s_{1}^{-1}/\sum_{i\;=\;1}^{n}s_{i}^{-1} & s_{2}^{-1}/\sum_{i\;=\;1}^{n}s_{i}^{-1} & \cdots & s_{n}^{-1}/\sum_{i\;=\;1}^{n}s_{i}^{-1}\\ \vdots & \vdots & \ddots & \vdots \\ s_{1}^{-1}/\sum_{i\;=\;1}^{n}s_{i}^{-1} & s_{2}^{-1}/\sum_{i\;=\;1}^{n}s_{i}^{-1} & \cdots & s_{n}^{-1}/\sum_{i\;=\;1}^{n}s_{i}^{-1} \end{array}\right]}_{N\times n}$$

(32) $$W_{j,k}=G_{j,k}\cdot S_{j,k}\;(j=1,2\cdots N,k=1,2\cdots n)$$

(33) $$K^{\prime }{}_{N\;\times \;1}={\left[\begin{array}{cccc} W_{1,1}/\sum_{i\;=\;1}^{n}W_{1,i} & W_{1,2}/\sum_{i\;=\;1}^{n}W_{1,i} & \cdots & W_{1,n}/\sum_{i\;=\;1}^{n}W_{1,i}\\ W_{2,1}/\sum_{i\;=\;1}^{n}W_{2,i} & W_{2,2}/\sum_{i\;=\;1}^{n}W_{2,i} & \cdots & W_{2,n}/\sum_{i\;=\;1}^{n}W_{2,i}\\ \vdots & \vdots & \ddots & \vdots \\ W_{N,1}/\sum_{i\;=\;1}^{n}W_{N,i} & W_{N,1}/\sum_{i\;=\;1}^{n}W_{N,i} & \cdots & W_{N,n}/\sum_{i\;=\;1}^{n}W_{N,i} \end{array}\right]}_{N\times n}{\left[\begin{array}{c} K_{1}^{\prime }\\ K_{2}^{\prime }\\ \vdots \\ K_{n}^{\prime } \end{array}\right]}_{n\times 1}$$

where *S* and *G* represent the reformed weights of the standard deviation (Equation (19)) and Gaussian distribution based on the distance, respectively. $s_{i}$ is the *i*th standard deviation of local phase-elevation ratio over the *i*th block. 

**Figure 2:**
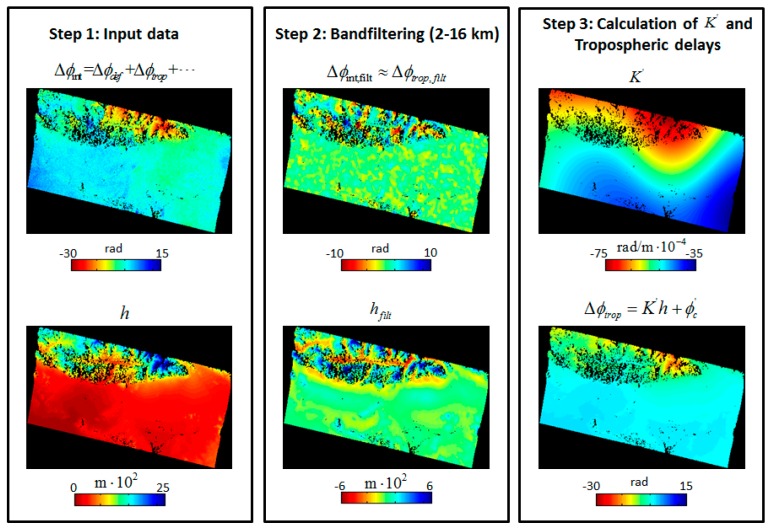
Tropospheric delay estimated using the RMW method for the interferogram created from images collected on 12 July 2008 and 18 April 2009. Step 1: the original interferometric phase $\Delta \phi_{\mathrm{int}}$ above and the elevation h below were derived from the SRTM DEM. Step 2: the band filtering in the 2–16 km spatial wavelength. Step 3: a spatial map of $K^{\prime }$ was acquired after local robust estimation for each block and subsequent extrapolation using multi-weights for all PS points. The final tropospheric delays (Step 3, lower figure) were obtained using the rewritten linear model (Equation (3)).

The multi-weight *W* is a N×n matrix. $K^{\prime }$ is the final derived phase-elevation ratio for all PS points. *N* is the number of PS points and n is the number of blocks for the whole interferogram. Figure 2 shows a step-by-step example of the RMW method for an interferogram spanning 12 July 2008 to 18 April 2009.

## 4. Results and Discussion

### 4.1. Time Series InSAR Results

**Figure 3:**
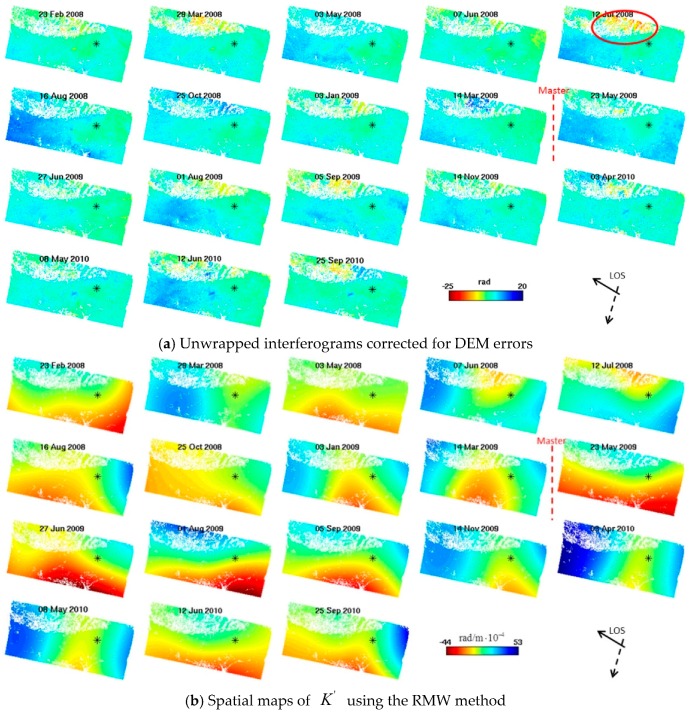

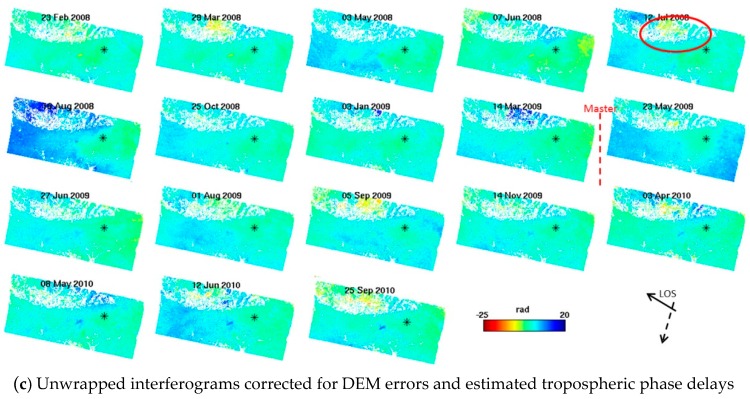
(**a**) Unwrapped time series interferograms corrected for DEM errors, containing tropospheric phase delays and deformation phases; (**b**) Spatial maps of *K*’ estimated by our RMW method; (**c**) Final unwrapped differential interferograms corrected for DEM errors and the tropospheric phase delays using our RMW method. The master date is 18 April 2009 and the asterisk represents the center of the reference area with a 300-m radius. A change of 2π radians corresponds to a 28 mm displacement in the LOS direction.

We used StaMPS software to perform InSAR time series analysis and to estimate DEM errors. After PS selection and DEM error correction, the interferograms were unwrapped in three dimensions [24]. The unwrapped time series interferograms after the DEM errors were removed are shown in Figure 3a. The signals in these unwrapped interferograms consist of the contribution of the ground deformation, which we are interested in understanding and the tropospheric phase delay that must be removed. Note that the spatial-temporal filtering in StaMPS is not used to estimate the tropospheric delay. Instead we used our RMW method to calculate the delays for individual interferograms. 

**Figure 4:**
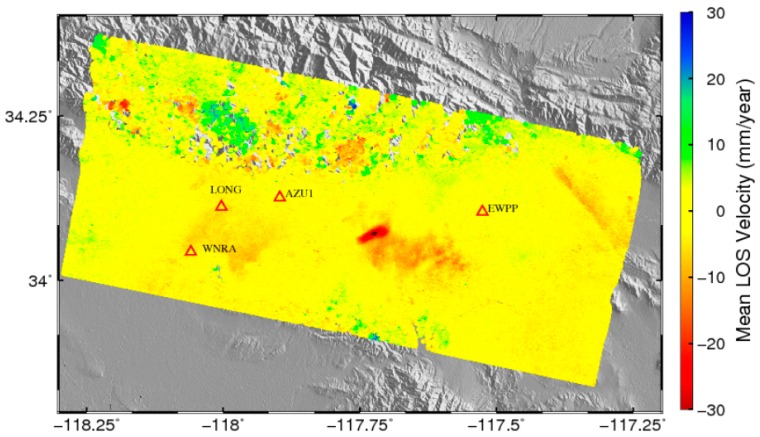
Mean displacement rates (mm/year) of PS in the Line of Sight (LOS) direction after removal of DEM errors and tropospheric phase delays in the study area (The red triangles represent the locations of GPS sites and the background is the SRTM DEM).

The spatial map of $K^{\prime }$ estimated from our RMW method is shown in Figure 3b. Figure 3c shows the interferograms that the tropospheric phase delay estimated by our RMW method has been subtracted. Finally, the mean-velocity map over the study area was obtained (Figure 4). Subsidence in the Pomona Basin can be observed in the unwrapped interfergrams (Figure 3a) and the mean-velocity map (Figure 4). 

Those interferograms in Figure 3a without tropospheric delay correction contain both ground deformation and tropospheric phase delay. We found that the phase delay partly varies with topography. For example, the interferogram for 12 July 2008 shows strong spatial correlation with surface topography. As Figure 3c shows, these delays were significantly mitigated after correction of the interferogram using our RMW method. We validated our RMW method by comparing with the conventional linear method. The linear method shown in Equation (1) assumes a simple phase-topography relationship for individual interferograms and therefore does not allow for spatially variable tropospheric delay. However, we were able to account for the spatial variation of tropospheric delay by applying the RMW method, as can be seen from the spatial variation maps of $K^{\prime }$ shown in Figure 3b. Here, we compared the mitigation results of the RMW method with the unwrapped interferograms, expecting a large reduction in local correlation to topography. In order to find a clear phase-topography relationship, we excluded pixels at an altitude below 800 m because of the deformation signal existing in the Pomona Basin. In this region, the unwrapped phases are variable and discontinuous in some areas and therefore the tropospheric signals are contaminated by deformation signals. These can be observed in the 8 May 2010, 12 June 2010, and 25 September 2010 interferograms shown in Figure 3a. It is still important to remove the contributions from deformation -contaminated bands over the flat area in Pomona Basin, especially in the area A (Figure 1) and its surrounding regions. The variable and discontinuous phases in the interferograms demonstrate tropospheric change and deformation mask influences. Therefore, a robust and spatially varying tropospheric delay correction is necessary.

**Figure 5:**
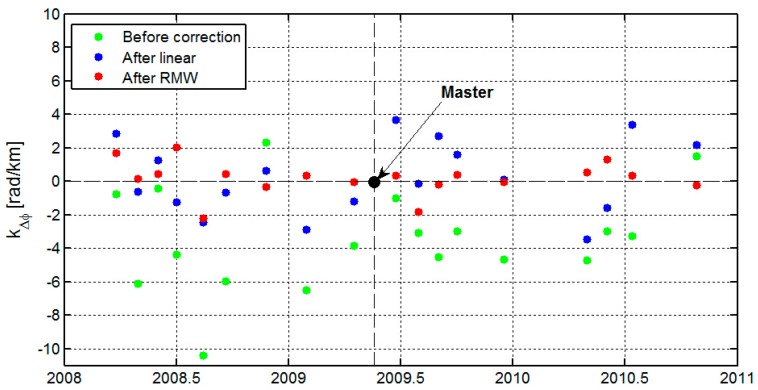
Comparing the local correlation $k_{\Delta \phi }$ between the interferogram Δϕ and topography h. Only pixels with altitude above 800 m are used. Where $\Delta \phi =k_{\Delta \phi }h\;+\;\phi_{c}$, then $k_{\Delta \phi }$ is estimated before (green points) and after correction for the tropospheric delays, using the conventional linear method (blue points) and the RMW method (red points). The constant $\phi_{c}$ represents an overall offset and was applied to the whole interferogram.

On average, we found a strong local relationship |*k*_Δ_*_φ_*| of 3.9 rad/km (~1.7 cm/km) for our 18 interferograms before the tropospheric correction (the green points shown in Figure 5). After the linear correction method (blue points), we found an average reduction in the local correlation to topography of 2.0 rad/km (~0.9 cm/km). However, the local correlation increased at an average value of 1.3 rad/km (~0.6 cm/km) in the 23 February 2008, 3 May 2008, 23 May 2009, 12 June 2010, and 25 September 2010 interferograms. 

**Figure 6:**
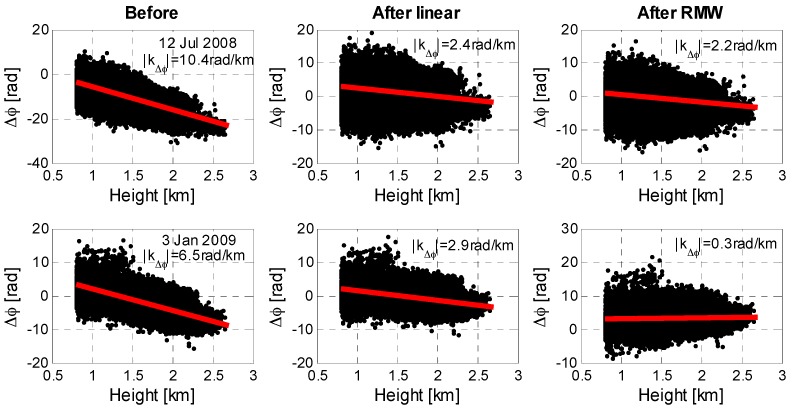
Scatterplots of the local correlation between interferograms and topography. A comparison of the estimated ratio *k*_Δ*φ*_ (red solid line) before and after correction for tropospheric delays using the linear and RMW methods for the 12 July 2008 and 3 January 2009 interferograms.

After treatment with the RMW method (red points), we found an average reduction in local correlation with topography of 3.1 rad/km (~1.4 cm/km), about three fourth of the signal, with the increase only for 23 February 2008 (0.9 rad/km or 0.4 cm/km) interferogram. The strongest reductions were observed in the 12 July 2008 (8.2 rad/km or 3.7 cm/km) and 3 January 2009 (6.2 rad/km or 2.8 cm/km) interferograms. Figure 6 shows the two interferograms with the strongest reduction results before and after the tropospheric corrections. These two interferograms show the reductions in local correlation with topography of 8.0 rad/km (~3.6 cm/km) and 3.6 rad/km (~1.6 cm/km) using the linear method. The RMW method however performs better, with reductions of 8.2 rad/km (3.7 cm/km) and 6.2 rad/km (2.8 cm/km). These comparative results show that the RMW method outperforms the conventional linear method. Additionally, taking the 23 February 2008 interferogram as an example, the local correlation increased 2.1 rad/km (~0.9 cm/km) while the correlation based on the full interferogram decreased 3.6 rad/km (~1.6 cm/km) after the linear correction. The increase in local correlation was due to the spatial variation of the troposphere, which cannot be captured using the linear method. Thus, the linear method estimated the phase-elevation ratio by a conventional LS method based on the whole interferogram and did not account for inconsistent filter bands and spatially variable tropospheric delays.

**Figure 7:**
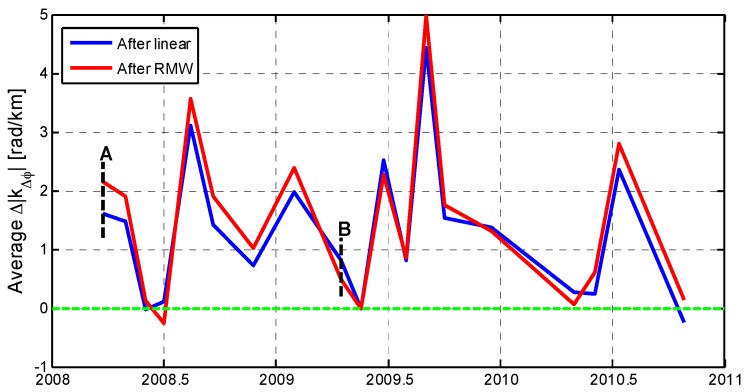
The difference value $\Delta \;|k_{\Delta \phi }|$ indicates a reduction or increase in local correlation with topography before and after tropospheric correction. This graph shows a comparison of the average $\Delta \;|k_{\Delta \phi }|$ in the center of 28 blocks for the corresponding interferogram after the correction for the linear (blue solid line) and RMW methods (red solid line). A comparison of A and B indicated by dashed black lines are specifically shown in Figure 8.

We further tested the advantages of our RMW method by splitting the interferograms into 28 blocks with different terrain patterns. Each block covers about 100 km^2^ containing mountain areas, basin areas, or both. We sampled the phases at the center of each block to examine the correlations between the original and the corrected phases, as shown in Figure 7. On average, we found a reduction (above zero) in local correlation with topography of 1.3 rad/km (~0.6 cm/km) except for the 3 May 2008 and 25 September 2010 interferograms after the linear method correction. After RMW correction, the local correlation decreased with an average value of 1.5 rad/km (~0.7 cm/km) for 17 out of 18 interferograms. The RMW method also performed better compared to the linear method with better reductions for 13 out of the total 18 interferograms.

**Figure 8:**
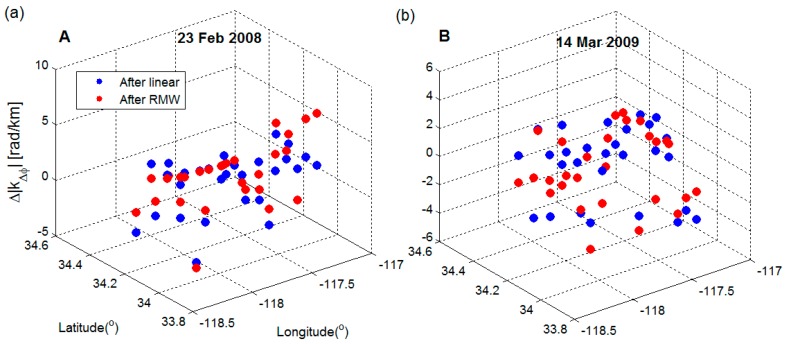
Comparison of the variation in the spatial spots of estimation $\Delta \;|k_{\Delta \phi }|$ after correction using the linear (blue solid points) and RMW methods (red solid points) for each block. The two examples; the (**A**) 23 February 2008 and (**B**) 14 March 2009 interferograms, show the largest differences of average $\Delta \;|k_{\Delta \phi }|$. Blocks cut across mountainous areas, basin areas, or both in segments of about 10 km.

Figure 8 shows the two largest differences of average $\Delta \;|k_{\Delta \phi }|$ between the two methods. For the 23 February 2008 interferogram (Figure 8a), we found that the RMW method decreased correlation with the topography to an average of 2.2 rad/km (~1.0 cm/km), 0.6 rad/km (~0.3 cm/km) an improvement over the linear method. For the RMW method, 21 blocks had an average reduction of 3.6 rad/km (~1.6 cm/km); while for other blocks, an average increase (below zero) of 2.0 rad/km (~0.9 cm/km) was observed. For the linear method, 20 blocks had an average reduction of 3.3 rad/km (1.5 cm/km) and an average increase of 2.8 rad/km (~1.3 cm/km), and less efficient than the results from our RMW method. For the 14 March 2009 interferogram (Figure 8b), the RMW method generally under-performed the linear method, the average increase was only 1.8 rad/km (~0.8 cm/km) while for the linear method the increase was 2.6 rad/km (~1.2 cm/km). As the RMW method is applied locally, we found that it is more subject to contamination from various tropospheric delay components present at different spatial scales. This leads to a biased estimation of phase- elevation ratios with an average value of 0.4 rad/km (~0.2 cm/km) larger than the linear method. However, the impact of turbulence on the linear method appears less severe than for the RMW method. Due to the spatially variable estimation in the RMW method, the average standard deviation of $\Delta \;|k_{\Delta \phi }|$ for all interferograms was 0.2 rad/km larger than the linear method, as can be seen from the variation in the spatial maps of $\Delta \;|k_{\Delta \phi }|$ shown in Figure 8. The RMW method accounts for spatial variation by applying multi-weighted phase-elevation ratios robustly estimated from reliable band-filtered data on 40 blocks of half overlaps.

In general, the band-pass components usually display a clearly linear relationship between phase and topography, unlike the high-pass and low-pass components. The high pass and low pass components are therefore excluded from the estimation of phase-elevation ratios. The bandwidth range is limited by the resolution of the data and spatial extent of our study region. We performed a statistical comparison of our final performance results using multiple spatial bands. These results show that spatial bands on the 2–4 km, 4–8 km, 8–16 km, 2–8 km and 2–32 km wavelengths have an average reduction in the local correlation with topography of 1.2 rad/km (~0.5 cm/km) with 0.1 rad/km (~0.1 cm/km) fluctuation. The 2–16 km spatial band showed an average reduction in the value for local correlation to topography of 1.5 rad/km (~0.7 cm/km). The 16–32 km bands performed less effectively, with an average reduction of 0.6 rad/km (~0.3 cm/km). Thus, the 2–16 km spatial band was less sensitive to the other signals, such as deformation, turbulence and residual orbit errors. However, the 2–16 km spatial band cannot completely remove the inconsistent bands and could cause residual signals to leak into the estimated tropospheric delay. However, the RMW method can significantly mitigate the effects of contaminated filter bands, but does not always work well. In some regions, phase does not seem linearly related to topography due to multiple tectonic influences or other non-tectonic sources and the variability of the atmospheric circulation due to turbulent tropospheric delays.

**Figure 9:**
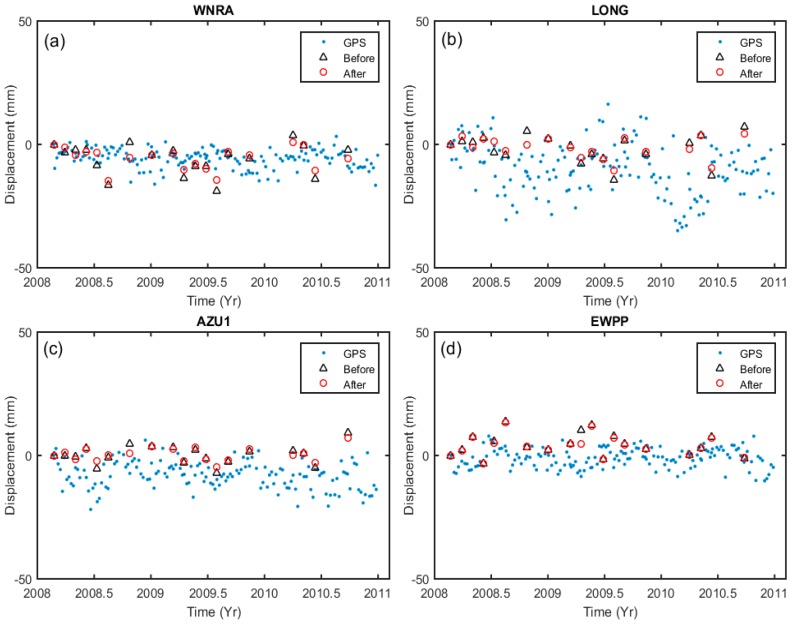
Shows a comparison of the LOS displacements measured by InSAR and GPS (blue points) at stations (**a**) WNRA; (**b**) LONG; (**c**) AZU1; and (**d**) EWPP. The black triangles and red circles represent displacements estimated from StaMPS with and without tropospheric correction, respectively.

In order to validate our results, we compared the time series of LOS displacements measured by GPS to displacements measured by InSAR. We used four GPS stations from the Southern California Integrated GPS Network (SCIGN) for our comparison. The North, East, and Up direction displacement from GPS were projected to LOS direction for comparison. In Figure 9, we show the time series plots of measurements from the four GPS stations and ground displacement as estimated from InSAR images. The black triangles in Figure 9 represent displacement as estimated from InSAR without tropospheric correction, and the red circles indicate the displacement where the tropospheric delay was mitigated using our proposed RMW method. Because the GPS stations are located on flat terrain, topographically correlated tropospheric effects are not significant. The RMS errors before tropospheric correction on GPS stations are WNRA (14.22) mm, LONG (18.55 mm), AZU1 (11.57 mm) and EWPP (7.65 mm), after correction the RMS error was reduced to 10.79 mm, 16.26 mm, 10.88 mm and 5.51 mm, respectively. These comparisons support our argument that the proposed RMW method is quite effective for mitigating the topographically correlated tropospheric delay and thus enables the extraction of more reliable surface deformation information.

### 4.2. The Sensitivity of RMW Method to Orbital Ramp

**Figure 10:**
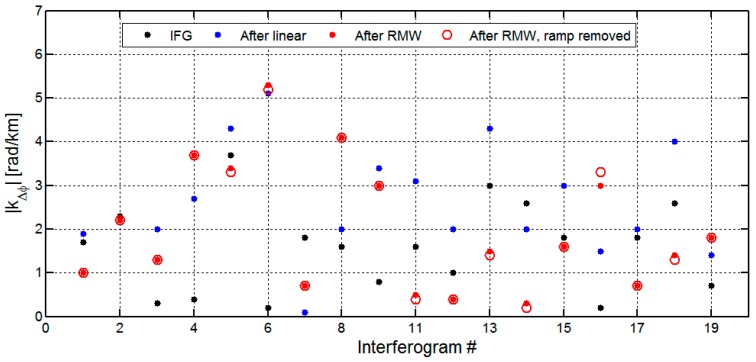
Comparison of the phase-elevation ratio $\left|k_{\Delta \phi }\right|$ for full interferograms after correction with the linear method (blue points), RMW method (red points) and with the ramp-removed RMW method (red circles). The comparative unwrapped interferograms (black points) were corrected for DEM and residual orbital errors, respectively.

In this section, we will discuss how the orbital ramp influences the estimate of the phase-elevation ratio $k_{\Delta \phi }$. To do this, we compare our results with the $k_{\Delta \phi }$ values derived from the RMW method with the ramp retained and removed (Figure 10). For the full interferogram, on average we found a reduction in local correlation with topography of 1.0 rad/km (~0.5 cm/km) for 11 interferograms after correction with our RMW method. Among these interferograms, the local correlation increased with an average value of 0.5 rad/km (0.2 cm/km) after the linear method correction. The RMW method performed more efficiently in comparison to the linear method with reductions of correlation in 10 out of 11 interferograms. To account for residual orbital errors, the original interferogram was corrected with a plane trend in the range and azimuth before applying our RMW method. Figure 10 illustrates that the local correlation estimated from the ramp-retained RMW method is almost the same as the local correlation estimated from the ramp-removed RMW method. This suggests that our RMW method is insensitive to the influence of the orbital ramp and therefore can estimate the tropospheric delay even when orbital errors or long wavelength deformation signals are present.

### 4.3. The Effects of Turbulent Delay

We also considered how turbulence mixing in the interferogram influenced estimates of $k_{\Delta \phi }$. The tropospheric delay is considered as the sum of two components, stratified delay and turbulent delay. The stratified delay results from different vertical refractivity profiles during two SAR acquisitions, showing a strong correlation with topography. This applies to mountainous terrain only. Turbulence is an irregular and random motion, and dependent on the random characteristics of meteorological phenomena affected by flat as well as mountainous terrain. The focus of our study is to estimate the stratified delay that often dominates the tropospheric signal in an interferogram. The analysis in Section 4.2 shows that for most inteferograms, the RMW method is quite effective in mitigating topographically correlated tropospheric delays. However, some cases show very small improvements and the RMW method may even introduce atmospheric estimation error, leading to inaccurate information in the final deformation results (Figure 10).

We selected two interferograms with a wider coverage to test the effect of turbulence effects (the blue rectangle in Figure 1). We validated our results using the passive multispectral imager Medium-Resolution Imaging Spectrometer (MERIS) onboard the Envisat satellite [35]. MERIS -derived prediction is restricted to instances where the cloud coverage is less than 20% of the scene. At the scale of an interferogram, the spatial variations in pressure are usually small, typically at an order of magnitude of 1 hPa. 

**Figure 11:**
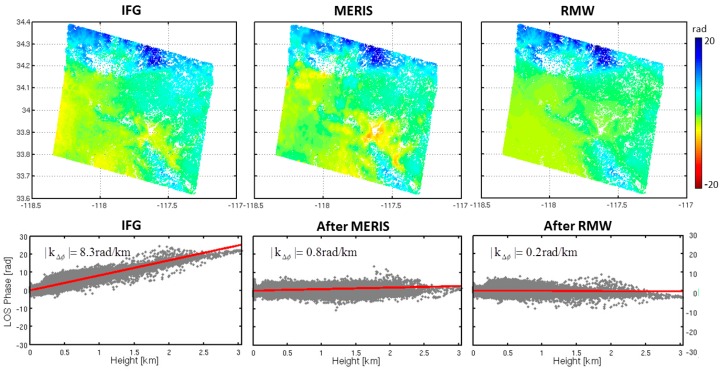
A comparison of the performance of the MERIS correction and the RMW method correction. A stratified dominant interferogram was generated using two SAR acquisitions on 16 August 2008 and 25 October 2008 by the Envisat satellite over California. The average *B*_⊥_ is 272 m. The original interferogram has been corrected for the residual orbital errors. The tropospheric delay has a strong linear relationship with elevation. The prediction of tropospheric delay using MERIS and the RMW method show a good agreement, with a difference in the standard deviation of 0.1 rad along the LOS. The standard deviation of the residuals after correction using MERIS data was about 1.6 rad and 1.7 rad after correction with the RMW method. The standard deviation reduction after correction with the RMW method was about 55%.

**Figure 12:**
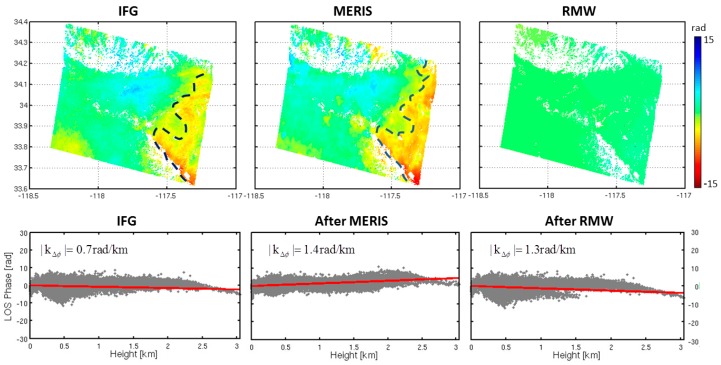
A comparison of the performance of the MERIS correction and the RMW method correction. A turbulent dominant interferogram was generated using two SAR acquisitions collected on 23 May 2009 and 27 June 2009. The average *B*_⊥_ is 406 m. The original interferogram was corrected for residual orbital errors. As shown, the troposphere delay is not linearly related to topography. The standard deviation reduced by about 42% after correction with MERIS while the standard deviation increased about 8% after correction with the RMW method.

We excluded the hydrostatic component when we calculated the MERIS tropospheric delay. When the stratified delays dominate signals (Figure 11), the tropospheric delay shows a strong correlation with topography, this delay can be accurately estimated based on the RMW method. Hence, the tropospheric effects are significantly mitigated. When turbulent delay overwhelmed the interferogram (Figure 12), we found that both the original interferogram and the prediction of tropospheric delay did not appear in the correlation between the phase and topography. The RMW method cannot properly estimate such small-scale tropospheric signal, shown on the right side of the dashed line in Figure 12. Therefore, our proposed RMW method is not suitable for estimating significant turbulence mixing effects in interferograms.

## 5. Conclusions

To tackle the issue of contaminated and inconsistent bands in the processing of phase-based tropospheric delay correction, we propose a novel expanded form of the robust estimation method for larger spatial data sets, RMW. As the analysis of experimental results show, we take advantage of the 2–16 km band-filter to estimate the spatially variable phase-elevation ratio *K* robustly; in a way that is relatively suitable and advantageous for areas with confounding processes. The method splits a region into multiple blocks (about 100 km^2^ in our study) and estimates the ratio *K* locally to resolve lateral variations in stratification over lager regions. The standard deviations derived by robust estimation combined with Gaussian weights were used to construct a multi-weighted factor function for spatially variable tropospheric correction. The multi-weighted factor function constructed with standard deviation simultaneously considers the robustness in the observation and structure spaces. The iterative calculation approach applied in the RMW method is relatively straightforward and efficient. In some regions of our study area, the proposed method was very effective when no other auxiliary and independent data was available.

We tested the RMW method over Southern California, where the mountainous areas and the flat area coexist with a small-scale deformation in the Pomona Basin. After correction, we found a better degree of reduction in the topographically correlated tropospheric signals than the conventional linear method, and an improved correlation between InSAR and GPS estimated surface displacements. We can mitigate bias in displacement rate estimates by robust estimated phases while using time series analysis in the differential interferograms. Experimental results also demonstrated that the RMW method is insensitive to orbital ramps. However, the differences between RMW method and conventional linear methods were relatively small in flat terrain areas. Our method is based on the same assumption made by earlier studies of a linear relationship between topography and phase; therefore some tropospheric phase components do not directly relate linearly to topography directly, given complex turbulent atmospheric delay effects and tectonic influences or effects stemming from other, non-tectonic sources. Therefore, in some cases, more comprehensive correction methods are required due to the complicated geological structure of the earth and the variability in the atmospheric circulation.

## Figures and Tables

**Table 1:** Parameters of Envisat ASAR images.

Ifg Index	Date	Perpendicular Baseline (m)	Temporal Baseline (Days)	Doppler Central Baseline (Hz)
1	23 February 2008	−95	420	9.6
2	29 March 2008	439	385	−1.9
3	3 May 2008	91	350	7.8
4	7 June 2008	308	315	0.1
5	12 July 2008	364	280	0.1
6	16 August 2008	284	245	4.1
7	25 October 2008	272	175	−3.4
8	3 January 2009	288	105	−0.4
9	14 March 2009	692	35	1.7
**10**	**18 April 2009**	**0**	**0**	**0**
11	23 May 2009	203	−35	6.0
12	27 June 2009	407	−70	−0.7
13	1 August 2009	98	−105	0.1
14	5 September 2009	432	−140	−3.7
15	14 November 2009	356	−210	−3.0
16	3 April 2010	543	−350	−7.5
17	8 May 2010	369	−385	3.7
18	12 June 2010	389	−420	−15.7
19	25 September 2010	391	−525	−10.6

**Table 2:** StaMPS processing parameters.

Parameter	Value	Parameter	Value
DEM (SRTM)	90 m	Gamma convergence	0.005
Oversample	no	Density random	20
Dispersion threshold	0.4	Weed STD	1
Patches number	6	Weed max noise	Inf
Max topography error	5	Unwrap method	‘3D’
Select method	Density	Unwrap grid size	200 m

**Table 3:** Improvement of standard deviation (STD) by the robust estimation.

Ifg Index	Improvement STD (%)	Ifg Index	Improvement STD (%)
1	23.7	11	6.8
2	5.4	12	2.4
3	0.2	13	13.1
4	4.2	14	3.0
5	3.7	15	8.4
6	1.6	16	6.0
7	14.4	17	14.0
8	2.4	18	12.8
9	0.1	19	28.7
